# Racial disparities in law enforcement/court-ordered psychiatric inpatient admissions after the 2008 recession: a test of the frustration–aggression–displacement hypothesis

**DOI:** 10.1007/s00127-024-02627-z

**Published:** 2024-02-20

**Authors:** Parvati Singh, Ralph Catalano, Tim A. Bruckner

**Affiliations:** 1 Division of Epidemiology, College of Public Health, The Ohio State University, 338 Cunz Hall, 1841 Neil Avenue, Columbus, OH 43210 USA; 2 Graduate School, Public Health, University of California, Berkeley, Berkeley, CA USA; 3 Department of Health, Society, and Behavior, University of California, Irvine, CA USA; 4 Center for Population, Inequality, and Policy, University of California, Irvine, CA USA

**Keywords:** Involuntary psychiatric commitments, African Americans, Economic recession, Racial disparities, Longitudinal analysis

## Abstract

**Background:**

Societies under duress may selectively increase the reporting of disordered persons from vulnerable communities to law enforcement. Mentally ill African American males reportedly are perceived as more threatening relative to females and other race/ethnicities. We examine whether law enforcement/court order-requested involuntary psychiatric hospitalizations increased among African American males shortly after ambient economic decline—a widely characterized population stressor.

**Methods:**

We identified psychiatric inpatient admissions requested by law enforcement/court orders from 2006 to 2011 across four US states (Arizona, California, New York, North Carolina). Our analytic sample comprises 13.1 million psychiatric inpatient admissions across 95 counties over 72 months. We operationalized exposure to economic downturns as percent change in monthly employment in a metropolitan statistical area (MSA). We used zero inflated negative binomial and linear fixed effects regression analyses to examine psychiatric inpatient admissions requested by law enforcement/court orders following regional employment decline over a time period that includes the Great Recession of 2008.

Findings.

Declines in monthly employment precede by one month a 6% increase in psychiatric hospitalizations requested by law enforcement/court order among African American males (*p* < 0.05), but not among other race/sex groups. Estimates amount to an excess of 2554 involuntary admissions among African American males statistically attributable to aggregate-level employment decline.

**Conclusions:**

Economic downturns may increase involuntary psychiatric commitments among African American males. Our findings underscore the unique vulnerability of racial/ethnic minorities during economic contractions.

**Supplementary Information:**

The online version contains supplementary material available at 10.1007/s00127-024-02627-z.

## Introduction

In the USA, persons perceived as dangerous to self, dangerous to others or gravely disabled may be hospitalized against their will for psychiatric treatment [1]. Such involuntary psychiatric hospitalizations, also referred to as civil commitments, require clinical assessment and often initiate from community-level reporting of mentally disordered persons to law enforcement and mental health professionals [2–4]. However, community-level identification of individuals for involuntary psychiatric hospitalization tends to reflect inherent biases against sub-groups regarded as threatening or disturbing to the well-being of other members [5]. During periods of heightened ambient stress, these biases may lead to over-reporting of some members, particularly from minority populations. This phenomenon, ascribed to the ‘frustration–aggression–displacement’ hypothesis, finds support in the literature wherein societies under duress may lower their tolerance of deviant and noisome behavior in general, and of sub-groups traditionally perceived as threatening or disruptive to social order, in particular [4, 6–10].

Economic contraction serves as a population-level stressor that may increase involuntary psychiatric hospitalizations [4, 6, 7]. Ecological research suggests that during economic downturns, populations may cope with economic uncertainty by moderating their own behavior and regulating their immediate environment [4]. Regulation of immediate environment at the population level may increase frustration, micro-aggression and aversion to bothersome or ‘noisome’ individuals, and lower social tolerance of the mentally ill, or of individuals whose behavior departs from the perceived social order [9–14]. Regulation of one’s own behavior, on the other hand, may correspond with lower aggression and greater impulse control [7, 10]. The ‘net effect’ of these mechanisms depends on the relative contribution and strength, at the population level, of frustration–aggression–displacement and self-regulation [7].

Research examining the ‘frustration–aggression–displacement’ hypothesis finds a pronounced increase in involuntary psychiatric hospitalization among males, following economic downturns [4, 7, 15]. This relation presumably arises from (1) societal perception of males as more threatening relative to females, and (2) greater labor market attachment of males relative to females that may elicit a stronger response to labor market contraction [16–18]. African American males, in particular, may face a higher risk of social punitive measures, including involuntary psychiatric hospitalizations, owing to stereotypical social perceptions of this group as more threatening relative to other race/ethnicities [8, 19, 20]. Seminal research finds elevated aggression toward African Americans in the form of lynching following decline in cotton prices in the late nineteenth and early twentieth century [21]. In the modern context, economic downturns may correspond with greater social micro-aggressions in that members of civil society may leverage socially sanctioned mechanisms to report and remove African American males through law enforcement authorities [4]. Catalano et al. [4] report a 5% increase in the number of involuntarily committed African American men, 2 months following increased unemployment in California, over a 13-year period spanning 1985–1998. Kessel et al. [15] also report a similar increase (~5%) in involuntary psychiatric holds among males within 3 weeks of increased unemployment insurance claims in Florida from 1999 to 2003.

Relative to involuntary psychiatric hospitalizations initiated by other sources (e.g., family members or mental health professionals), those requested by law enforcement/court orders may specifically indicate racial scapegoating through displaced frustration and aggression [15]. It is plausible that a contracting economy may increase the number of disordered persons who meet the clinical criteria for involuntary psychiatric hospitalization. The relative incidence, however, of such hospitalizations should not be expected vary by source of initiation if the incidence of disorder increased uniformly [15]. Empirical research, however, does not support this expectation. By contrast, the literature finds that law enforcement-initiated psychiatric hospitalizations increase during economic downturns even after controlling for those initiated by mental health providers [15]. Researchers attribute this increase to reduced tolerance in the population that in turn, may reflect strong social biases [15].

In this study, we examine whether law enforcement/court order-requested involuntary psychiatric hospitalizations of African American men increase during economic contractions. We build upon prior work [4, 15], to test whether population rates of law enforcement/court order-requested involuntary psychiatric hospitalizations among African American men increase within 0–3 months of aggregate macroeconomic decline. In alignment with prior work, we use an exposure window of 0–3 months to permit sufficient time for economic downturns to induce population-level behavioral and psychiatric changes, while also maintaining a relatively brief temporal lag to limit confounding from other (temporally stable) factors [4, 15, 22]. We examine four US states (Arizona, California, New York, North Carolina) and exploit regional variation in economic contractions over a time period (2006–2011) that includes the Great Recession of 2008 [4, 15].

## Methods

### Data and variables

We retrieved data on psychiatric inpatient admissions for select states from the State Inpatient Database (SID). The SID provides a near-census of all hospital inpatient admissions for participating states and is made available for purchase by the Agency for Healthcare Research and Quality under the Healthcare Cost Utilization Project (HCUP) [23]. SID reports individual admission-level diagnoses (ICD 9 codes) for all inpatients. We include all inpatients with a psychiatric diagnosis for mental illnesses listed within the Clinical Classification Software (CCS) categories [24] (Appendix Table A.1 of Supplementary Material). Some states also allow the SID to report whether a psychiatric admission was requested by law enforcement/court order (coded under admission source or point of origin) [24]. Among the states that participated in HCUP SID from 2006 to 2011, California, Arizona, North Carolina and New York report admission source (including law enforcement/court order), race, gender, county identifier and admission month. The most populous state in this study—California—does not provide information on race beyond 2011, hence, we restrict our analysis to 2011. These states comprise the study regions in our analysis and yield a total of 13.1 million psychiatric inpatient admissions from 2006 to 2011.

We identified 46,188 law enforcement/court order-initiated involuntary psychiatric inpatient admissions within the 13.1 million ‘universe’ of all psychiatric admissions. The SID does not directly report whether a psychiatric inpatient admission was voluntary or involuntary. We approximate this status based on whether law enforcement (or a court order) requested the admission, in keeping with the medico-legal definitions of voluntary/involuntary status of inpatient psychiatric admissions in the USA [25–27]. Two variables in SID, namely *asource* (admission source) and *pointoforigin* (point of origin), directly reported from the Uniform Billing form (UB04)—which is the standard claim form for billing medical and mental health claims—record whether a patient’s inpatient admission was requested by a law enforcement authority and/or a court order [28–31]. Psychiatric inpatient admissions that were transported to the hospital by emergency law enforcement responders do not receive a ‘requested by law enforcement/court order’ status on the UB04 form and in the SID [31]. This status, also referred to as a patient’s ‘legal status’ helps identify involuntary versus voluntary admissions as patients voluntarily admitted for psychiatric treatment largely originate from *non*-law enforcement/court order admission sources or point of origin (e.g., emergency room, another hospital, other health facility, routine, etc.) [25–27, 32]. Other studies use *asource* and *pointoforigin* variables for examining characteristics of psychiatric inpatient admissions and quality of care in the SID [33, 34]. Our observed count of 46,188 law enforcement/court order-initiated involuntary psychiatric admissions aligns with expected counts reported nationally by the Substance Abuse and Mental Health Services Administration and hospital/facility count in the SID [35, 36].^1^

We excluded all psychiatric inpatient admissions with missing information for race, sex, month, admission source (or point of origin) or county (~10% of total observations). Our final analytic sample comprised 25,640 county-months (95 counties over 72 months, with four race and sex sub-groups per county-month). Of these, 6866 county-months reported one or more psychiatric inpatient admissions requested by law enforcement/court order.

Male African American psychiatric inpatients admitted through a request from law enforcement/court order form the main group of interest in our analysis. We aggregated the monthly count of psychiatric inpatient admissions per county over 72 months (January 2006–December 2011) by race (African American, all other races), sex (male, female) and admission status (requested by law enforcement/court order, all other admission types). We converted the monthly counts of psychiatric admissions to rates per 100,000 population using county-level race and gender population denominators from the US Census Bureau’s Population Estimates database [37]. The resulting series of monthly counts and population rates (by race, sex, admission type) form our outcome variables.

We operationalized, as our exposure, the percent change in monthly employment per metropolitan statistical area (MSA). MSAs comprise large, heavily populated urban centers of economic activity that may span one or more counties [38, 39]. MSA-level employment, measured monthly in the Current Population Survey (CPS) by the US Bureau of Labor Statistics, provides the total number of people who worked for pay (either part time or full time) during the survey reference week [38]. Employment change, defined as the difference in a month’s number of employed persons from the previous month divided by the previous month’s total employed people, gives acute changes in a local economy (i.e., MSA) in that it is zero (or of negligible value) if there is very little change in number of employed people, but high (either negative or positive) in circumstances of abrupt economic decline or expansion. Employment change accounts for changes in the civilian labor force and is well suited for modeling economic recession as ‘shocks’. Month-to-month variation in employment change overcomes the drawbacks of other macroeconomic indicators such as unemployment rate, mass layoffs and foreclosures as (a) it is not limited to only those eligible for unemployment insurance, (b) represents immediate change, accounts for inflows, outflows or changes in the civilian labor force and (c) does not present directional distortion during brief periods of economic expansion [40–45]. Its suitability as a strong, acute indicator of macroeconomic contraction is further evidenced by its use in literature documenting associations between economic downturns and health outcomes [22, 40, 46, 47]. We obtained monthly aggregated employment series from 2006 to 2011, for each metropolitan statistical area (MSA) within our study states, from the Local Area Unemployment Statistics database made available by the US Bureau of Labor Statistics [38]. We merged MSA-level data to SID counties using MSA-to-county crosswalk made available by the National Bureau of Economic Research [48]. Our final analytic sample comprised 46 MSAs (spanning 95 counties in four states) over 72 months (2006–2011).

### Analysis

We hypothesize that the psychiatric inpatient admissions requested by law enforcement/court order increases among African American men, but not among other race/gender groups, in the months immediately following a decline in percent monthly employment change. Additionally, we examine whether monthly employment decline precedes changes in *non*-law enforcement/court order-requested inpatient psychiatric admissions among African American men and other race/ethnicity and sex groups. We use two analytic approaches based on count versus population-standardized rate outcome distributions to determine the relation between our outcomes and exposure (modeled as 0- to 3-month exposure lags):

1. Zero inflated binomial (ZINB) regression: This approach examines the relation between county-level monthly *counts* of psychiatric inpatient admissions requested by law enforcement/court order (outcome) and 0- to 3-month lags of monthly employment change (exposure). ZINB models accommodate overdispersion and excessive zeros by combining a negative binomial distribution with a separate process for excess zeros (in our case, logistic regression) [49, 50]. In epidemiologic research, ZINB models tend to fare better than other approaches when excess zeros and non-zero counts in the outcome variable arise from different data generating processes, respectively (e.g., hurdle models that deem all zeros as structural, rather than a combination of structural and sampling zeros) [51]. In addition to the 0- to 3-month exposure lags, our ZINB models controlled for population, month, year fixed effects and state-specific linear time trends.
2. Fixed effects ordinary least squares (OLS) regression: This approach uses, as the outcome, the county-level log-transformed *population rate* [i.e., natural logarithm of (counts/population)*100,000] of psychiatric inpatient admissions requested by law enforcement, controlling for county, month, year fixed effects and state-specific linear time trends [22]. Fixed effects OLS regression incorporates fixed effects for individual counties, capturing unobserved heterogeneity, and allows for the control of time-invariant characteristics, permitting the estimation of within county relation between the exposure and outcome over time [52].

We estimate separate regressions per admission type (requested by law enforcement/court order, all other psychiatric admissions), by race (African American, non-African American) and sex (male, female). In alignment with prior work, we contend that absent an increase in *non*-law enforcement/court order-requested psychiatric inpatient admissions, any observed rise in law enforcement-requested involuntary admissions among African American men following ambient macroeconomic decline would support the frustration–aggression–displacement hypothesis [4, 15]. Sensitivity checks include re-estimation of our main tests (with psychiatric inpatient admissions requested by law enforcement/court order as the outcome), controlling for all other psychiatric admissions, to account for potential confounding from overall changes in psychiatric help seeking following exposure. For tests that reject the null, we estimate the predicted counts of inpatient psychiatric admissions with incremental change in exposure. We use Stata’s ‘margins’ command to compute and graph average marginal predicted counts of psychiatric inpatient admissions (per unit increase in exposure) for race/sex groups and admission types that reject the null [53]. We conduct all analyses in Stata SE (version14.2) [54].

## Results

Table 1 describes psychiatric inpatient admissions and percent monthly employment change in our study sample. Psychiatric inpatient admissions requested by law enforcement/court order are highest among African American males, followed by African American females. The same holds for psychiatric inpatient admissions *not* requested by law enforcement/court orders although the magnitude of these admission types are greater than the former owing to the rare nature of law enforcement-initiated admissions.

**Table 1:** Descriptive statistics of psychiatric inpatient admissions by race, sex and type (requested by law enforcement/court order, *not* requested by law enforcement/court order) and percent monthly employment change across 95 counties (within 46 MSAs, AZ, CA, NC, NY) over 72 months (2006–2011)

Variables	Counts (mean, SD)
Psychiatric inpatient admissions (counts) requested by law enforcement/court order per county-month among	
African American males	7076 (mean = 1.17, SD = 3.55)
African American females	3032 (mean = 0.51, SD = 2.66)
Non-African American males	23,830 (mean = 3.50, SD = 12.68)
Non-African American females	12,250 (mean = 1.79, SD = 3.55)

	Mean	SD	Range (min)	Range (max)
Psychiatric inpatient admissions (per 100,000 population) requested by law enforcement/court order per county-month among				
African American males	7.03	2.92	2.18	15.22
African American females	2.18	1.59	0.54	9.95
Non-African American males	1.35	0.96	0.39	6.56
Non-African American females	0.80	0.97	0.16	5.92
Psychiatric inpatient admissions (per 100,000 population) *not* requested by law enforcement/court order per county-month among:				
African American males	416.64	37.87	350.96	528.77
African American females	403.82	38.70	319.48	501.49
Non-African American males	262.63	19.30	224.64	301.92
Non-African American females	286.88	20.96	241.15	328.59
Percent monthly employment change per MSA-month	−0.02	1.26	−10.18	10.45

*SD* standard deviation

**Fig. 1:**
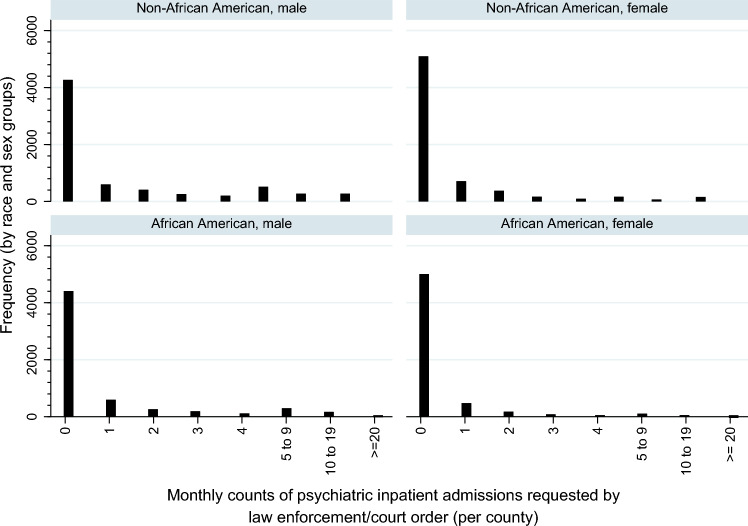
County-level monthly counts of psychiatric inpatient admissions requested by law enforcement/court order, by race (African American, non-African American) and sex (male, female), across 46 MSAs (AZ, CA, NC, NY), 2006–2011

Figure 1 shows the frequency distribution of county-level monthly law enforcement/court-ordered psychiatric inpatient admission counts by race and sex groups in our data. All groups show high frequency of zero monthly counts and high dispersion, supporting our utilization of zero inflated negative binomial regression approach. Appendix Figure A.1 of Supplementary Material shows the frequency distribution of non-law enforcement/court-ordered psychiatric inpatient admissions in our sample.

**Fig. 2:**
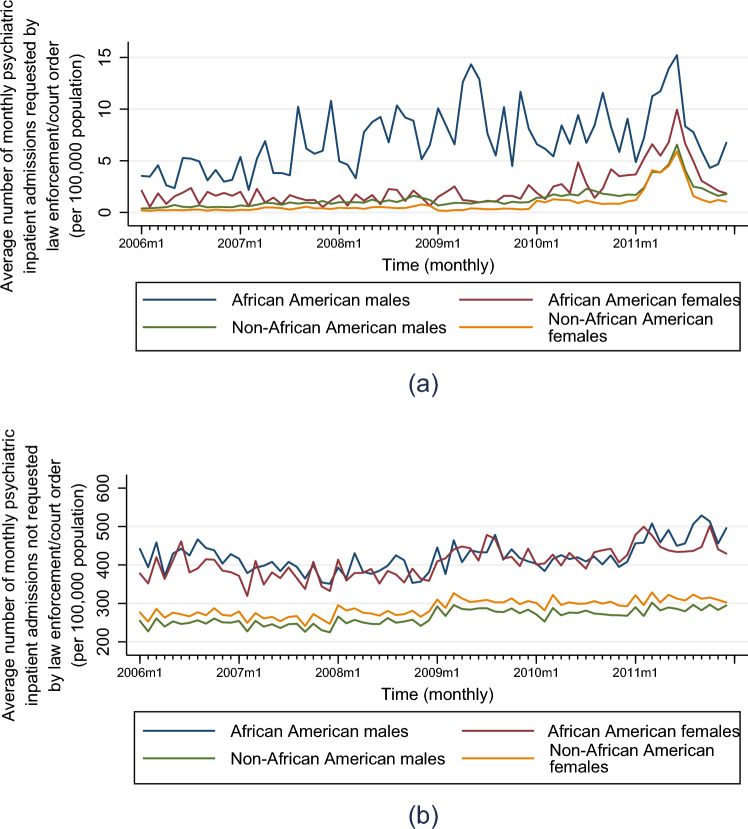
Average county-level psychiatric inpatient admissions (per 100,000 population) per month requested by law enforcement/court order (**a**) and *not* requested by law enforcement/court order (**b**), by race (African American, non-African American) and sex (male, female), across 46 MSAs (AZ, CA, NC, NY), 2006–2011

Temporal trends in law enforcement/court order-requested psychiatric inpatient admission rates (per 100,000 population) show substantially higher rates among African American males relative to all other groups (Fig. 2a). Among psychiatric hospitalizations *not* requested by law enforcement/court order, African Americans exhibit higher rates than non-African Americans but males and females (within African Americans) show similar admission rates over time (Fig. 2b).

**Fig. 3:**
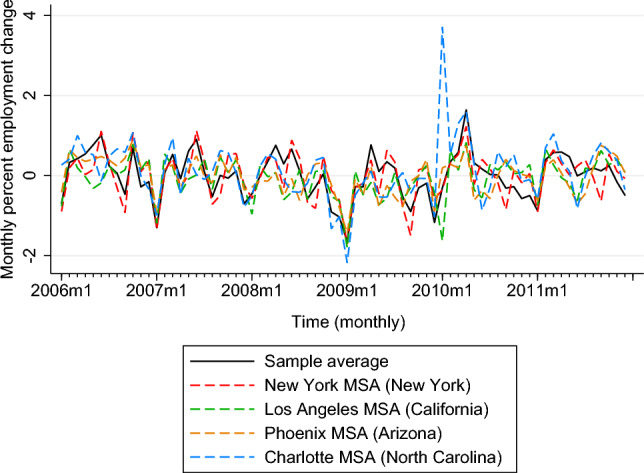
Average monthly percent employment change across (1) the full sample comprising 46 MSAs (AZ, CA, NC, NY), (2) New York MSA (NY), (3) Los Angeles MSA (CA), (4) Phoenix MSA (AZ) and (5) Charlotte MSA (NC), 2006–2011

Figure 3 graphs the average monthly percent change in MSA-level employment across the 46 MSAs included in this study as well as in the largest MSA in each study state (Los Angeles MSA—California, New York MSA—New York, Phoenix MSA—Arizona, and Charlotte MSA—North Carolina). Here, negative values indicate employment decline and positive values show aggregate increase in employment (relative to previous month). The sharpest aggregate decline in employment levels occurs in January 2009 for the full sample (on average) as well as in the four largest MSAs in study states. Notably, Charlotte Metropolitan Area (North Carolina) shows rapid increase in employment levels post 2009 (in January 2010).

Table 2 presents the results from zero inflated negative binomial regression analysis of monthly counts of psychiatric inpatient admissions requested by law enforcement/court order as a function of percent monthly employment change (lag 0–3). For a one-unit increase in percent employment change, the incidence rate of psychiatric hospitalizations requested by law enforcement/court order among African American males increases by a factor of 0.06 1 month later, i.e., at exposure lag 1 (Table 2, model a). We do not observe statistically detectable associations between exposure lags and outcome for other race/sex groups (Table 2, models b, c, d). Results from Table 2 (model a) suggest a 6% increase in the risk of law enforcement/court order-requested psychiatric inpatient admissions among African American males, following a one percent decline in exposure.

**Table 2:** Zero inflated negative binomial (ZINB) regression results predicting the incidence rate ratio (IRR) of monthly counts of psychiatric inpatient admissions requested by law enforcement/court order as a function of percent monthly employment change (0- to 3-month lags) and other covariates (population offset, year, month fixed effects, state-specific linear time trends; not shown for simplicity)

Negative binomial component of ZINB regression predicting number of psychiatric inpatient admissions requested by law enforcement/court order								
	African American				Non-African American			
	Model a: males		Model b: females		Model c: males		Model d: females	
Exposure	IRR	95% CI	IRR	95% CI	IRR	95% CI	IRR	95% CI
Percent monthly employment change Lag 0	0.93	0.91,1.02	0.95	0.90,1.01	0.98	0.97,1.01	0.99	0.96,1.04
Percent monthly employment change Lag 1	0.94**	0.92,0.98	0.97	0.90,1.04	0.99	0.97, 1.02	1.01	0.96,1.05
Percent monthly employment change Lag 2	0.98	0.93,1.03	0.96	0.90,1.02	0.99	0.97, 1.03	1.00	0.96,1.05
Percent monthly employment change Lag 3	1.02	0.97,1.07	1.00	0.95,1.06	0.99	0.95, 1.02	1.02	0.97,1.07

Logistic component of ZINB regression predicting zero psychiatric inpatient admissions requested by law enforcement/court order								
	Coeff	95% CI	Coeff	95% CI	Coeff	95% CI	Coeff	95% CI
*Zero inflation covariates*								
Percent monthly employment change Lag 0	0.01	−0.10,0.11	−0.01	−0.12,0.11	−0.02	−0.08,0.04	0.16***	0.07,0.25
Percent monthly employment change Lag 1	0.00	−0.07,0.07	0.04	−0.09,0.17	0.02	−0.04,0.07	0.04	−0.07,0.15
Percent monthly employment change Lag 2	−0.04	−0.13,0.06	0.07	−0.06,0.19	−0.02	−0.11,0.07	0.04	−0.07,0.14
Percent monthly employment change Lag 3	−0.03	−0.11,0.05	−0.01	−0.15,0.14	−0.04	−0.10,0.02	−0.01	−0.10,0.08
Sample size (county-months)	6055		5953		6803		6829	
Non-zero observations	1645		949		2536		1736	
Zero observations	4410		5004		4267		5093	

Standard errors clustered by metropolitan statistical area

*95% CI* 95% confidence interval

* *p* value <0.1, ** *p* value <0.05, *** *p* value <0.01, **** *p* value <0.001

Table 3 presents the results from linear fixed effects regression analysis of (log-transformed) psychiatric inpatient admissions requested by law enforcement/court order (per 100,000 population) as a function of percent monthly employment change (lag 0 to 3). Whereas this analysis excludes all observations with 0 values of the outcome and thus reflects a weaker test of our hypothesis, results from Table 3 qualitatively cohere with those from our main test in Table 2. Decline in percent employment change precedes by 1 month an increase in psychiatric hospitalizations requested by law enforcement/court order among African American males but not among other race/sex groups. Here, a one-unit decline in percent monthly employment corresponds with 0.03 unit increase in (log-transformed) psychiatric admissions one month later (Table 3, model a). Put another way, law enforcement/court order-requested psychiatric inpatient admissions (per 100,000 population) among African American males increase by 3%, following a one percent decline in exposure.

**Table 3:** Linear fixed effects regression results predicting (log-transformed) psychiatric inpatient admissions (per 100,000 population) requested by law enforcement/court order as a function of percent monthly employment change (0- to 3-month lags) (other covariates not shown; controls include county, year, month fixed effects and state-specific linear time trends)

Exposure	African American				Non-African American			
	Model a: Males		Model b: Females		Model c: Males		Model d: Females	
	Coeff	95% CI	Coeff	95% CI	Coeff	95% CI	Coeff	95% CI
Percent monthly employment change Lag 0	−0.026	−0.06, 0.003	−0.005	−0.03, 0.02	−0.010	−0.03, 0.01	0.001	−0.02, 0.02
Percent monthly employment change Lag 1	−0.032**	−0.06, −0.01	0.005	−0.02, 0.03	−0.005	−0.03, 0.02	0.010	−0.02, 0.04
Percent monthly employment change Lag 2	0.007	−0.02, 0.03	0.002	−0.02, 0.02	−0.003	−0.02, 0.02	0.008	−0.02, 0.04
Percent monthly employment change Lag 3	0.004	−0.02, 0.03	0.023	−0.003, 0.05	0.001	−0.02, 0.02	0.010	−0.02, 0.04
Sample size (county-months)	1645		949		2536		1736	

Standard errors clustered by metropolitan statistical area

*95% CI* 95% confidence interval

* *p* value <0.1, ** *p* value <0.05, *** *p* value <0.01, **** *p* value <0.001

We did not fit zero inflated negative regression models for counts of *non*-law enforcement/court-ordered psychiatric admissions as this series did not meet the requisite criteria for ZINB regression analysis (no zeros observed in monthly counts, see Appendix Figure A.1 of Supplementary Material), and examined this variable (as the outcome) using its log-transformed population rate (per 100,000 population). Among psychiatric admissions *not* requested by law enforcement/court order, we fail to reject the null for all race and sex groups at all exposure lags (Appendix Table A.2 of Supplementary Material).

As a sensitivity check for shared trends between the two admission types, we replicated the analyses from Tables 2, 3 and include *non*-law enforcement/court-ordered psychiatric admissions as a covariate (Appendix Tables A.3, A.4 of Supplementary Material). Inference from these analyses remains unchanged relative to Tables 2 and 3.

To give the reader a sense of the magnitude of psychiatric admissions requested by law enforcement/court orders statistically attributable to decline in exposure, we estimated the predicted counts of admissions in this group using Stata’s *margins* routine following ZINB estimation [53]. Appendix Figure A.2 of Supplementary Material shows the predicted counts of psychiatric admissions requested by law enforcement/court orders among African American males versus non-African American males per unit increase in monthly percent employment change (lag 1) (obtained from zero inflated negative binomial regression analysis). We applied the average increase in predicted count (=6%, per unit decline in percent employment) to the mean rate of psychiatric admissions requested by law enforcement/court orders among African American males (=7.03 per 100,000 population) over 6055 county-months (that report these admissions among African American males). This exercise yields, for the state-years analyzed, an additional 2554 admissions (=7.03*6055*0.06) among African American males statistically attributable to aggregate employment decline.

## Discussion

During economic downturns, societies may exhibit reduced tolerance of deviant behavior [4, 9]. This reduction in tolerance, following macroeconomic contraction, may manifest as frustration–aggression–displacement behaviors through racial scapegoating of African American males to authorities for involuntary hospitalization. We examined this relation for four US states over 72 months, which include the 2008 recession. Psychiatric inpatient admissions requested by law enforcement/court orders increase among African American males (but not in other groups) one month following aggregate employment decline in a metropolitan statistical area. Additional sensitivity tests also show that this increase does not extend to *non*-law enforcement/court-ordered psychiatric admissions in African American men.

Key strengths of this study include the monthly resolution of exposure and outcome and examination of multiple geographies in the USA. Prior studies that motivated the present research are restricted to small areas (or single states) and predate the 2008 recession [4, 15]. We utilize datasets that capture nearly all psychiatric inpatient admissions on a monthly basis for four US states (Arizona, California, North Carolina, New York) and report whether these admissions were requested by law enforcement/court orders in a uniformly coded manner. Our analysis also supports temporal order such that the exposure precedes the outcome, and rules out confounding due to reverse causation as well as from long-run sequelae of economic downturns. We also control for inherent spatial and temporal attributes that may correspond with trends in psychiatric admissions using county, month and year fixed effects, and state-specific linear time trends. Our estimate of a 6% increase in law enforcement/court-ordered psychiatric admissions 1 month following aggregate employment decline aligns with other studies that find a 5% increase in these visits among males in general [15] and African American men in particular [4], over the same temporal window.

Limitations include that we do not distinguish between (1) danger to others from danger to self and grave disability within psychiatric admissions requested by law enforcement/court orders, and (2) other types of involuntary psychiatric admissions, such as those requested by mental health professionals. This circumstance stems from the data limitation that SID does not report reason for admission and whether an involuntary psychiatric admission was requested by other (*non*-law enforcement/court order) authorities or family members. However, this delineation may not be strictly necessary for testing the frustration–aggression–displacement hypothesis as prior studies emphasize psychiatric admissions requested by law enforcement/court orders to gauge social tolerance [15]. We also recognize the potential for underestimation of “true” relation between employment decline and law enforcement/court-ordered psychiatric inpatient admissions owing to MSA-level analysis wherein counties within MSAs may exhibit differentially higher or lower responses during economic recessions. Our analyses utilize employment trends in MSAs as these regions form large, urban centers of concentrated economic activity, and we encourage future research to build upon this work once monthly data on employment become available at the county level.

This study is also limited with respect to identification of new versus repeat inpatient psychiatric admissions owing to lack of data on patient histories in the SID database. Economic stressors, inherent during recessions, may serve as catalysts for an augmented incidence or relapse of severe mental disorders, particularly affecting African Americans disparately [41]. The unique challenges faced by African Americans communities, such as systemic inequities and historical disparities, may intensify the impact of economic hardships on mental health outcomes [55]. Given the elevated likelihood of police involvement and involuntary admissions in pathways to mental health care within African Americans communities, our study’s findings may arise from selective increase in law enforcement-mediated psychiatric help seeking for this demographic. Whereas we do not find an increase in *non*-law enforcement/court order-requested psychiatric admissions in our analyses, mental health care for African Americans remains fraught with systemic biases and discriminatory practices and may exacerbate the vulnerability of this population during periods of macroeconomic contractions.

Our results, in combination with those from prior research, underscore the lack of mental health care access among African Americans [55]. This group exhibits lower access to primary mental health care services and relies extensively on emergency departments for psychiatric care owing to lack of insurance, greater stigma, discrimination within the health care system, low quality of treatment and higher likelihood of misdiagnosis [55, 56]. These barriers are more pronounced for African American men who tend to exhibit lower psychiatric help seeking relative to African American women during recessions [57]. These systemic ‘gaps’ and unmet needs in mental health care may expand during economic downturns when public health agencies face funding shortages and may be forced to reduce supply.

Ambient shocks associated with violence or hostility may also increase racial scapegoating through frustration–aggression–displacement mechanisms against vulnerable groups. Research finds that in Florida, law enforcement-initiated involuntary psychiatric examinations increased in the weeks immediately following the 9/11 terrorist attacks [58]. It is plausible that such exogenous shocks, that either increase social fear or promote targeting of ethnic minorities, may correspond with higher involuntary psychiatric commitments of the targeted groups. We encourage future research to examine whether involuntary psychiatric commitments increase following such macrosocial shocks.

## Conclusion

Societal responses to ambient macroeconomic shocks may place disproportionately higher disadvantages on African Americans. In this study, we focused on law enforcement/court-ordered psychiatric admissions to gauge the extent to which this outcome, which potentially corresponds with social frustration, changes during economic contractions. We find that these inpatient admissions increase selectively among African American men, 1 month following aggregate employment decline, but do not exhibit statistically detectable changes in other race/sex groups. The results from this study highlight the unique vulnerability of African American communities and motivate the development of targeted interventions for mental health support, economic opportunities, community outreach and law enforcement training, in service of African Americans.

## Supplementary Information

Below is the link to the electronic supplementary material.

Supplementary file1 (DOCX 37 KB)

## Data Availability

Data used in this study are available for purchase from the Healthcare Cost & Utilization Project (HCUP), State Inpatient Database (SID). URL: https://hcup-us.ahrq.gov/sidoverview.jsp

## Footnotes

1 SAMHSA [36] reports a total of 82,914 involuntary psychiatric inpatient admissions across 10,374 psychiatric-related care facilities in 2010 in the USA (in SAMHSA [36]: see Table 1, Table 1a, Table 2a). This yields an average annual number of such admissions per facility = 8. Number of facilities (hospital IDs) reported in our study sample in SID (for AZ, CA, NC, NY) = 884 (AHRQ, 2014). Extrapolating over our study period of 6 years in our sample, crude 6-year estimate of involuntary psychiatric inpatient admissions = 8*884*6 = 42,392.
